# Variation of mesophyll conductance mediated by nitrogen form is related to changes in cell wall property and chloroplast number

**DOI:** 10.1093/hr/uhae112

**Published:** 2024-02-22

**Authors:** Yiwen Cao, Yonghui Pan, Yating Yang, Tianheng Liu, Min Wang, Yong Li, Shiwei Guo

**Affiliations:** Jiangsu Provincial Key Lab for Organic Solid Waste Utilization, National Engineering Research Center for Organic-based Fertilizers, Jiangsu Collaborative Innovation Center for Solid Organic Waste Resource Utilization, Nanjing Agricultural University, Nanjing, Jangsu, China; Jiangsu Provincial Key Lab for Organic Solid Waste Utilization, National Engineering Research Center for Organic-based Fertilizers, Jiangsu Collaborative Innovation Center for Solid Organic Waste Resource Utilization, Nanjing Agricultural University, Nanjing, Jangsu, China; Jiangsu Provincial Key Lab for Organic Solid Waste Utilization, National Engineering Research Center for Organic-based Fertilizers, Jiangsu Collaborative Innovation Center for Solid Organic Waste Resource Utilization, Nanjing Agricultural University, Nanjing, Jangsu, China; Jiangsu Provincial Key Lab for Organic Solid Waste Utilization, National Engineering Research Center for Organic-based Fertilizers, Jiangsu Collaborative Innovation Center for Solid Organic Waste Resource Utilization, Nanjing Agricultural University, Nanjing, Jangsu, China; Jiangsu Provincial Key Lab for Organic Solid Waste Utilization, National Engineering Research Center for Organic-based Fertilizers, Jiangsu Collaborative Innovation Center for Solid Organic Waste Resource Utilization, Nanjing Agricultural University, Nanjing, Jangsu, China; National Key Laboratory of Crop Genetic Improvement, Ministry of Agriculture Key Laboratory of Crop Ecophysiology and Farming System in the Middle Reaches of the Yangtze River, College of Plant Science and Technology, Huazhong Agricultural University, Wuhan, Hubei, China; Jiangsu Provincial Key Lab for Organic Solid Waste Utilization, National Engineering Research Center for Organic-based Fertilizers, Jiangsu Collaborative Innovation Center for Solid Organic Waste Resource Utilization, Nanjing Agricultural University, Nanjing, Jangsu, China

## Abstract

Plants primarily incorporate nitrate (NO_3_^−^) and ammonium (NH_4_^+^) as the primary source of inorganic nitrogen (N); the physiological mechanisms of photosynthesis (*A*) dropdown under NH_4_^+^ nutrition has been investigated in many studies. Leaf anatomy is a major determinant to mesophyll conductance (*g*_m_) and photosynthesis; however, it remains unclear whether the photosynthesis variations of plants exposed to different N forms is related to leaf anatomical variation. In this work, a common shrub, *Lonicera japonica* was hydroponically grown under NH_4_^+^, NO_3_^−^ and 50% NH_4_^+^/NO_3_^−^. We found that leaf N significantly accumulated under NH_4_^+^, whereas the photosynthesis was significantly decreased, which was mainly caused by a reduced *g*_m_. The reduced *g*_m_ under NH_4_^+^ was related to the decreased intercellular air space, the reduced chloroplast number and especially the thicker cell walls. Among the cell wall components, lignin and hemicellulose contents under NH_4_^+^ nutrition were significantly higher than those in the other two N forms and were scaled negatively correlated with *g*_m_; while pectin content was independent from N forms. Pathway analysis further revealed that the cell wall components might indirectly regulate *g*_m_ by influencing the thickness of the cell wall. These results highlight the importance of leaf anatomical variation characterized by modifications of chloroplasts number and cell wall thickness and compositions, in the regulation of photosynthesis in response to varied N sources.

## Introduction

Nitrogen (N) plays a crucial role in facilitating plant growth and development. In an agricultural ecosystem, the rational application of nitrogen fertilizer may be an efficient strategy to increase nitrogen use efficiency and decrease environmental pollution [1]. Higher plants utilize N from the soil mainly in the forms of ammonium (NH_4_^+^) and nitrate (NO_3_^−^) [2]. However, plant N form preference and uptake rates of different N forms are species-specific. Some plants, such as rice and tea plants showed strong preference to NH_4_^+^; whereas other species, such as *Arabidopsis thaliana* prefer NO_3_^−^ [3]. Even for NH_4_^+^-preferred plants, a supplement of NO_3_^−^ to NH_4_^+^ nutrition can lead to a better growth performance than plants supplied with NH_4_^+^ nutrition alone [4]. When NH_4_^+^ is supplied as the dominant or the only N source, plant growth is often inhibited, manifested by a reduced leaf area, relative growth rate and photosynthesis [2].

Over the past years, numerous studies have observed the influence of N form on leaf photosynthesis [5–7], but the mechanisms have not been fully understood. Mesophyll conductance (*g*_m_), stomatal conductance (*g*_s_), and biochemical capacity have been demonstrated to be three major limitations to leaf photosynthesis [8], and CO_2_ diffusion capacity contributes 35%–70% of total limitation to photosynthesis although the contribution can vary significantly at different environments [9]. However, it is not known which limitation is the major determinant to the response of photosynthesis to N form.

*g*_m_ represents the diffusion conductance of CO_2_ from substomatal cavities to carboxylation sites inside chloroplasts. *g*_m_ is a trait with high plasticity, and environmental stimuli including light intensity, water availability, salinity, and temperature can significantly affect *g*_m_ [10–13]. There is much evidence showing that *g*_m_ can vary substantially among different nutrient supplements [14–17]. For example, potassium deficiency can lead to more than 50% decline of *g*_m_ in *Brassica napus* leaves when comparing with normal potassium supplement [14]. Xiong *et al*. [17] indicated that *g*_m_ is sensitive to leaf N status, and the value of *g*_m_ increased with leaf N content. The importance of N source on *g*_m_ has also been intensively studied. Generally, leaf N content was positively correlated with net photosynthetic rate (*A*) and *g*_m_ [18]. Nevertheless, the response of *g*_m_ to different N forms exhibited variability in previous studies. Gao *et al*. [19] and Li *et al*. [20] showed a higher *g*_m_ under NH_4_^+^ conditions than that under NO_3_^−^ conditions in rice when subjected to drought stress. In contrast, Liu *et al*. [16] observed a lower *g*_m_ in female *Populus cathayana* when utilizing NH_4_^+^ as the sole N source. The regulatory mechanism underlying the relationship between *g*_m_ and N form remains unknown.

Leaf anatomical traits, including chloroplast surface area exposing to the intercellular air spaces (*S*_c_/*S*) and the thickness of cell wall (*T*_cw_), are major determinants to *g*_m_ [21]. *g*_m_ is positively correlated with *S*_c_, while *g*_m_ decreases substantially as *T*_cw_ increases [22, 23]. A meta-analysis by Huang *et al*. [24] suggested that *T*_cw_ shows great potential for enhancing leaf photosynthesis because of the large impact of *T*_cw_ on *g*_m_ and photosynthesis. The primary components of plant cell wall consist of cellulose microfibrils, along with a matrix of hemicelluloses, pectin, lignin, and structural proteins [25]. There are many studies reporting the large responses of cell wall characteristics to environmental stimuli, including drought, salinity, and temperature [26–28]. Transcriptomic analysis has highlighted a pivotal influence of N form on cell wall synthesis [29], which has been concluded from the co-expression of nitrate transporters and enzymes relating to cell wall synthesis. Unfortunately, the effects of inorganic N form on cell wall thickness and compositions are not fully understood.

To the best of our knowledge, there are merely two research studies indicating the potential impact of N form on cell wall properties. Podgórska *et al*. [30] found that, in comparison with NO_3_^−^ fed *Arabidopsis*, NH_4_^+^ fed plants possessed more densely assembled cellulose microfibrils and thicker cell walls, which in turn increased cell wall stiffness and inhibited plant growth. Głazowska *et al*. [31] proposed that NO_3_^−^ can stimulate cellulose while impeding lignin accumulation in *Brachypodium distachyon*; furthermore, structures of hemicellulose and pectins had been found to be strongly influenced by N form, and NO_3_^−^ can induce changes in the substitution pattern of xylan and result in reduced esterification level of homogalacturonan. However, the direct relationship between cell wall compositions and *g*_m_ were not addressed here. The influence of N form on the structure of cell wall and compositions can potentially affect photosynthesis *via g*_m_.

*Lonicera japonica*, commonly known as Honeysuckle, is a common shrub belonging to the Caprifoliaceae family [32], and it is an important Chinese medicinal material for its anti-inflammatory effect on viral infections [32]. In our previous study, *L. japonica* showed a preference to NO_3_^−^ in comparison with NH_4_^+^, which can be revealed from the rapid growth rate under NO_3_^−^ nutrition [33]. However, it is not known whether N form can substantially affect leaf photosynthesis in *L. japonica*. In this study, *L. japonica* was hydroponically grown with three N forms (NH_4_^+^ alone, 50%/50% NH_4_^+^/NO_3_^−^, and NO_3_^−^ alone). The aims of this research were: (i) to quatify and rank the *A* limitations factors under different N forms; (ii) to investigate the effect of N form on the leaf anatomical traits in determining *g*_m_, which include chloroplast development, cell wall thickness and compositions; and (iii) to explain the impact of N form on photosynthesis *via* leaf anatomical changes. The findings in this study will provide a theoretical basis for N management in *L. japonica*.

**Table 1:** Effects of different N forms on leaf photosynthetic characteristics in *Lonicera japonica*

Treatment	*A* (μmol m^−2^ s^−1^)	*g*_s_ (mol m^−2^ s^−1^)	*g*_m_ (mol m^−2^ s^−1^)	*V*_cmax_ (μmol m^−2^ s^−1^)	*J*_max_ (μmol m^−2^ s^−1^)	*C*_i_ (μmol mol^−1^)	*C*_c_ (μmol mol^−1^)
A	14.3 ± 0.4b	0.23 ± 0.02b	0.13 ± 0.01b	85 ± 2b	115 ± 3b	277 ± 3a	162 ± 6b
AN	18.1 ± 1.1a	0.35 ± 0.04a	0.25 ± 0.03a	98 ± 4a	141 ± 6a	275 ± 5a	202 ± 8a
N	19.9 ± 0.2a	0.35 ± 0.00a	0.26 ± 0.01a	103 ± 6a	157 ± 7a	267 ± 1a	189 ± 2a

*A*, net photosynthetic rate; *C*_c_, chloroplast CO_2_ concentration; *C*_i_, intercellular CO_2_ concentration; *g*_m_, mesophyll conductance; *g*_s_, stomatal conductance; *J*_max_, maximum electron transfer rate; *V*_cmax_, maximum carboxylation rate. A, AN, and N represent the N form of NH_4_^+^ alone, a mixed N supply, and NO_3_^−^ alone, respectively. Data are means ± SE (*n* = 5). The data followed by different letters are significant at *P* < 0.05 level.

## Results

### Effect of different N forms on leaf photosynthetic characteristics

**Figure 1:**
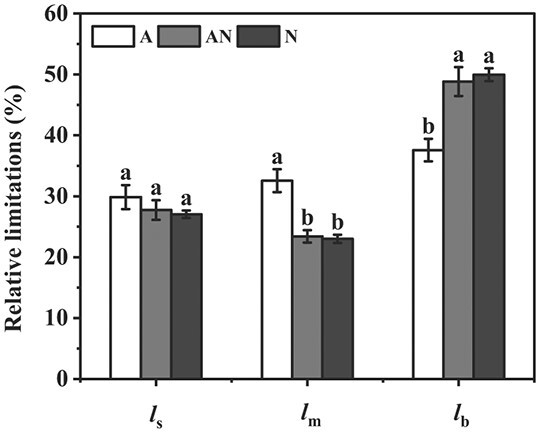
Relative limitation analyses of stomatal conductance (*l*_s_), mesophyll conductance (*l*_m_), and biochemical capacity (*l*_b_) on photosynthesis under different nitrogen(N) forms. A, AN, and N represent N forms of NH_4_^+^ alone, a mixed N supply, and NO_3_^−^ alone, respectively. The limitation of *l*_s_, *l*_m_, and *l*_b_ together add up to 100% at each treatment. Data are mean ± SE (*n* = 5).

There were considerable variations in leaf photosynthetic characteristics among different N forms. In general, leaf photosynthesis in NH_4_^+^ supplied plants were lower than those supplied with NO_3_^−^ or a mixed N supply of NH_4_^+^ and NO_3_^−^ (Table 1). Compared with NO_3_^−^ supply, *A*, *g*_s_, *g*_m_, maximum carboxylation rate (*V*_cmax_), and maximum electron transfer rate (*J*_max_) of NH_4_^+^ supplied plants were reduced by 28.1%, 36.4% 50.0%, 17.4%, and 26.7%, respectively. Leaf *A* was positively correlated with *g*_s_, *g*_m_, *V*_cmax_, and *J*_max_ among the treatments (Fig. S1, see online supplementary material). Intercellular CO_2_ concentration (*C*_i_) did not show any significance among the three treatments, but chloroplast CO_2_ concentration (*C*_c_) was notably more reduced in NH_4_^+^ treatment than that in the other two treatments (Table 1). In accordance with the lower *C*_c_ under NH_4_^+^ nutrition, the greater depressions observed in *g*_s_ and *g*_m_, compared to *V*_cmax_ and *J*_max_ suggested that the decline of CO_2_ diffusion conductance, especially of *g*_m_, was the major reason for the lowered photosynthesis in NH_4_^+^ nutrition than the other two N forms. This conclusion can also be drawn from the relative limitation analyses (Fig. 1). In general, the relative limitation of mesophyll diffusion on photosynthesis (*l*_m_) was larger in NH_4_^+^ nutrition (36.6%) than those in NO_3_^−^ (28.1%) and mixed N nutrition (26.6%); in contrast, the relative limitation of biochemical capacity on photosynthesis (*l*_b_) was lower in NH_4_^+^ nutrition (29.8%) than those in NO_3_^−^ (38.8%) and mixed N nutrition (41.8%). The relative limitation of stomatal conductance on photosynthesis (*l*_s_) was similar among three N forms.

### Effect of different N forms on leaf morphological and anatomical traits

**Figure 2:**
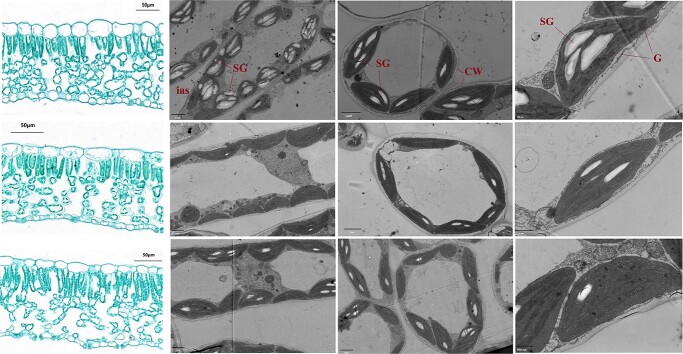
Light micrographs (a, e, and i, scale bar = 50 μm) and transmission electron micrographs (b, f, j, c, g, and k, scale bar = 2 μm; h, scale bar =1 μm; d and l, scale bar = 800 nm) of *Lonicera japonica* leaves exposed to different N forms. NH_4_^+^ alone (a–d), a mixed N supply (e–h), and NO_3_^−^ alone (i–l). C: chloroplast; CW: cell wall; G: grana; Ias: intercellular air space; SG: starch granule.

Compared to NO_3_^−^ and the mixed N supply, NH_4_^+^ nutrition significantly decreased *L*_A_ of the newly expanded leaves (Table S1, see online supplementary material), and N form had no substantial impact on leaf mass per area (*M*_A_), thickness (*T*_L_), or mesophyll thickness (*T*_mes_). In contrast, leaf ultrastructural characteristics were significantly affected by N form (Table 2 and Fig. 2). Obviously, mesophyll cells tended to be stacked together in plants fed with NH_4_^+^ nutrition and the starch granules were significantly accumulated in the chloroplasts (Fig. 2). Mesophyll cell surface area exposed to intercellular airspace (*S*_mes_/*S*), *S*_c_/*S*, chloroplast surface exposed to mesophyll cell surface area (*S*_c_/*S*_mes_), as well as the volume fraction of intercellular air space (*f*_ias_) were notably reduced in NH_4_^+^ treatment than those in the other two N forms; while *T*_cw_ and distance between adjacent chloroplasts (*D*_chl-chl_) were significantly higher under NH_4_^+^ treatment. N form had no significant effect on chloroplast length (*L*_chl_), chloroplast thickness (*T*_chl_), or distance between chloroplasts and cell walls (*L*_cyt_), which suggested that chloroplast size was independent on N form and that the variations of *S*_c_/*S* and *S*_c_/*S*_mes_ may relate to a different chloroplast number (Table 2; Table S2, see online supplementary material).

**Table 2:** Leaf anatomical characteristics of *Lonicera japonica* under different N forms

Treatment	*S*_mes_/*S* (m^2^ m^−2^)	*S*_c_/*S* (m^2^ m^−2^)	*S*_c_/*S*_mes_ (m^2^ m^−2^)	*f*_ias_ (%)	*T*_cw_ (μm)	*D*_chl-chl_ (μm)	*L*_cyt_ (μm)	*N*_chl1_ (No.)	*N*_chl2_ (No.)
A	11.4 ± 0.4b	6.16 ± 0.21b	0.54 ± 0.01b	20.2 ± 1.5b	0.253 ± 0.002a	1.08 ± 0.07a	0.21 ± 0.01a	7.7 ± 0.6c	4.9 ± 0.5b
AN	13.8 ± 0.4a	8.96 ± 0.20a	0.68 ± 0.02a	33.4 ± 0.3a	0.222 ± 0.002b	0.72 ± 0.02b	0.20 ± 0.01a	9.4 ± 0.4b	5.0 ± 0.4b
N	12.8 ± 0.4a	9.44 ± 0.43a	0.74 ± 0.02a	37.0 ± 2.3a	0.162 ± 0.002c	0.84 ± 0.03b	0.19 ± 0.02a	16.9 ± 0.1a	6.7 ± 0.1a

*D*_chl–chl_, the distance between adjacent chloroplasts; *f*_ias_, the volume fraction of intercellular air space; *L*_chl_, the distance between chloroplasts and cell walls; *N*_chl1_, chloroplast numbers in spongy parenchyma per mesophyll cell profile; *N*_chl2_, chloroplast numbers in palisade cell per mesophyll cell profile; *S*_c_/*S*, chloroplast surface area exposed to intercellular air space per leaf area; *S*_c_/*S*_mes_, chloroplast surface exposed to mesophyll cell surface area per leaf area; *S*_mes_/*S*, mesophyll cell surface area exposed to intercellular airspace per unit leaf area; *T*_cw_, cell wall thickness. A, AN, and N represent N forms of NH_4_^+^ alone, a mixed N supply, and NO_3_^−^ alone, respectively. A, AN, and N represent N forms of NH_4_^+^ alone, a mixed N supply, and NO_3_^−^ alone, respectively. Data are means ± SE (*n* = 4). The data followed by different letters are significant at *P* < 0.05 level.

**Figure 3:**
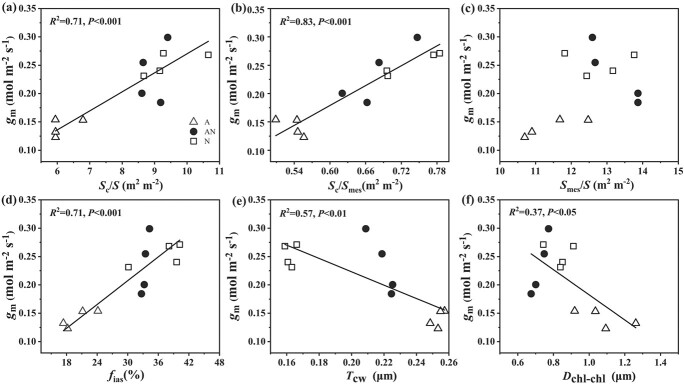
Correlations of *g*_m_ and chloroplast surface area exposed to intercellular air space per leaf area, *S*_c_/*S* (**a**), chloroplast surface exposed to mesophyll cell surface area per leaf area, *S*_c_/*S*_mes_ (**b**), mesophyll cell surface area exposed to intercellular airspace per unit leaf area, *S*_mes_/*S* (**c**), the volume fraction of intercellular air space, *f*_ias_ (**d**), cell wall thickness, *T*_cw_ (**e**), and the distance between adjacent chloroplasts, *D*_chl-chl_ (**f**). A, AN, and N represent treatment with NH_4_^+^ alone (open triangles), a mixed N supply (closed circles), and NO_3_^−^ alone (open squares), respectively. Data are fitted by linear regression (*n* = 4).

Indeed, the chloroplasts number in each cross section of spongy parenchyma and palisade cell was largely more decreased in NH_4_^+^ treatment than that in the other two N forms (Table 2), and chloroplast number was positively correlated with *S*_c_/*S* and *S*_c_/*S*_mes_ (Fig. S2, see online supplementary material). Moreover, *g*_m_ exhibited a positive correlation with *S*_c_/*S*, *S*_c_/*S*_mes_ and *f*_ias_, and a negative correlation with *T*_cw_ and *D*_chl-chl_ (Fig. 3). However, *g*_m_ did not show any significant correlation with *S*_mes_/*S*. These results suggested that the variation of *g*_m_ among the three N forms was related to the changes of leaf anatomical characteristics. This conclusion was also supported by the positive correlation between *g*_m_-Harley and *g*_m_-anatomy, which was calculated with leaf anatomical characteristics using gas diffusion model proposed by Tomás *et al*. [34] (Fig. S3, see online supplementary material).

### Key structural factors regulating *a* and *g*_m_

**Figure 4:**
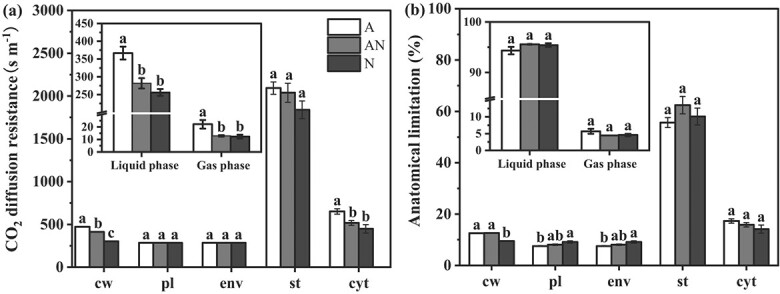
Anatomical limitations of mesophyll conductance (*g*_m_) (a) and the share of overall *g*_m_ limitation (b) by cell wall (cw), plasmalemma (pl), chloroplast envelope (env), stroma (st), and cytoplasm (cyt) under different N forms. The inset showed the anatomical limitations of *g*_m_ and the share of the overall *g*_m_ limitation by gas phase and liquid phase. A, AN, and N represent N form of NH_4_^+^ alone, a mixed N supply, and NO_3_^−^ alone, respectively. Data are means ± SE (*n* = 4). Different letters indicated a significance at *P* < 0.05 level.

**Figure 5:**
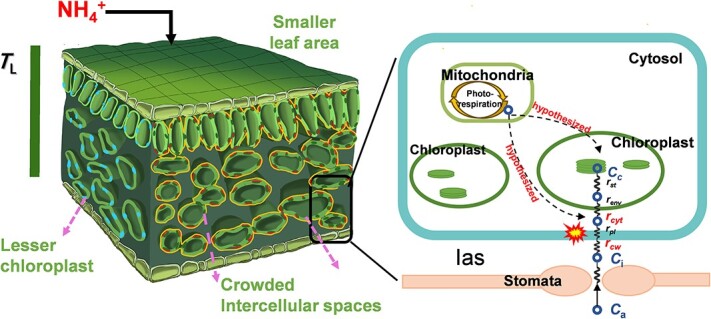
Schematic diagram of *Lonicera japonica* leaf anatomical traits (left side) and CO_2_ diffusion pathway under NH_4_^+^ alone supply (right side). In the left side, *S*_c_/*S* was outlined with red lines and *S*_mes_/*S* was marked with yellow lines. The accurate *S*_c_/*S* estimations overlapped *S*_mes_/*S* lines, whereas they are displayed independently here for clarity. Chloroplasts were highlighted with blue. Decreased chloroplast numbers resulted in the reductions in *S*_c_/*S*. The intercellular spaces were pointed out with pink discontinuous lines. The tight arrangement of mesophyll cells under sole NH_4_^+^ supplies led to increased intercellular spaces, which exacerbated the reductions in *S*_c_/*S* and *S*_mes_/*S*. In the right side, the black folded lines represent the strength of CO_2_ diffusion resistance into the cell from cell wall (*r*_cw_), plasma (*r*_pl_), cytoplasm (*r*_cyt_), envelope (*r*_env_), and stroma (*r*_st_), while the *r*_cw_ and *r*_cyt_ which are marked with red indicated the values of this component differed among the treatments (*P* < 0.05). Black discontinuous lines indicated the hypothesized pathways that could partially influence the CO_2_ diffusion and thereby *g*_m_.

Considering the different components along mesophyll diffusion pathway, the percentage limitations of *g*_m_ were estimated. Liquid-phase CO_2_ resistance was the predominant factor determining *g*_m_ (more than 90%) under different treatments (Fig. 4a), as CO_2_ diffusion rate in water was much slower than that in air. Among the components that were responsible for liquid-phase limitations, the stroma had the largest impact (55%–62%) on *g*_m_, but it was not statistically different among the treatments. In contrast, it seems that cell wall was the pivotal factor that differentiates the treatments, the cell wall resistance in NH_4_^+^ treatment was 56.3% larger compared to NO_3_^−^ treatment (Fig. 4b). Taken together, the impact of different forms of N on leaf morphological and anatomical structural factors were summarized in a schematic diagram (Fig. 5).

### N form had significant effects on cell wall compositions

**Figure 6:**
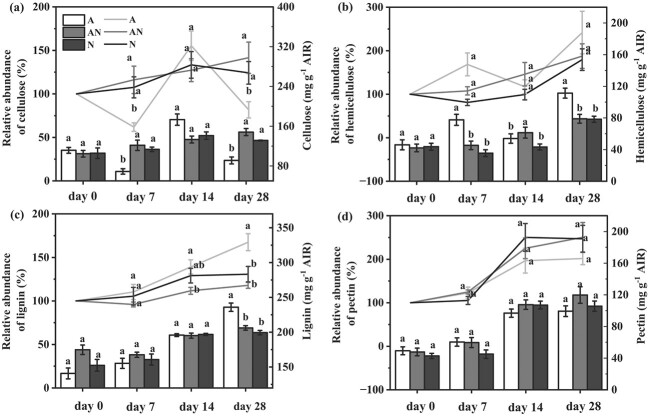
The absolute and relative abundance of cellulose (**a**), hemicellulose (**b**), lignin (**c**), and pectin (**d**) on day 0, day 7, day 14, and day 28 under different N forms. The histogram plot indicates the absolute abundance of cell wall components and the insert line chart represents the relative abundance of cell wall components compared with day 0. The amounts of specific cell wall components were expressed based on the amount of alcohol-insoluble residue (AIR). A, AN, and N represent the N form of NH_4_^+^ alone, a mixed N supply, and NO_3_^−^ alone, respectively. Data are means ± SE (*n* = 5). Different letters indicate a significance at *P* < 0.05 level.

In the present study, dynamic sampling of leaves fed with different N forms allowed us to investigate the detailed alterations in cell wall composition. Leaf photosynthesis and the cell wall components were continuously monitored since the start of N form treatment at an interval of 7 days (Fig. 6). Significant changes in absolute concentrations of cell wall compositions were detected since day 7. In general, NH_4_^+^ reduced cellulose content but increased hemicellulose level; nonetheless, N form supply did not have any significant effect on pectin abundance. Lignin content was significantly increased in NH_4_^+^ nutrition at 28 days after treatments, compared with NO_3_^−^ and mixed N treatments. Concerning the temporal variation in cell wall compositions among treatments, the relative abundance of each component was calculated based on day 0 (100%) (Fig. 6). The application of NO_3_^−^ and mixed N treatments resulted in a gradual increase of the relative abundance of all cell wall compositions since 7 days after the start of N form treatment. Whereas NH_4_^+^ supplement led to large fluctuations in the levels of cellulose and hemicellulose on day 7, where its relative abundance was statistically different from treatments of N and AN.

The relative abundance of lignin was notably higher compared to the N and AN treatments since day 14. *g*_m_ was positively correlated with cellulose concentration (Fig. S4, see online supplementary material, *R*^2^ = 0.46, *P* < 0.01), whereas it exhibited a negative correlation with both hemicellulose (Fig. S4, see online supplementary material, *R*^2^ = 0.49, *P* < 0.01) and lignin (Fig. S4, see online supplementary material, *R*^2^ = 0.57, *P* < 0.001). However, *g*_m_ was not correlated with pectin.

### Path analysis of the correlations between leaf anatomical characteristics and leaf photosynthesis

**Figure 7:**
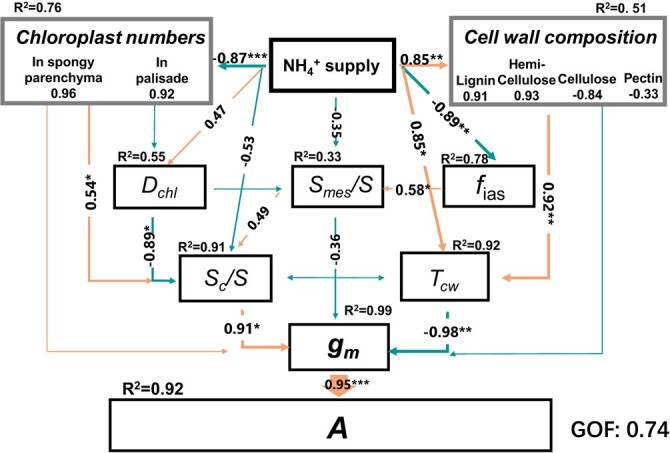
Path analysis showing the effects of the main leaf anatomy structures of *Lonicera japonica* induced by NH_4_^+^ application on *g*_m_ and net photosynthesis rate (*A*) (GOF = 0.74). Single-headed arrows indicate the hypothesized direction of causation, and the arrow width is proportional to the strength of the causal relationship. Orange arrows indicate a positive effect, whereas blue arrows indicate a negative effect. Numbers adjacent to arrows are path coefficients of the directly standardized effect size of the relationship, which are only shown for significant effects. The *R*^2^ values denote the proportions of variance explained by relationships with other variables in the model. The cell wall composition (day 28) and chloroplast numbers were divided into its composite variables; numbers adjacent to those variables are their coefficients to the composite variables. ^*^P < 0.05, ^**^P < 0.01, ^***^P < 0.001.

N form had a significant effect on leaf cell wall compositions, *T*_cw_, *f*_ias_, and chloroplast numbers. Path analysis indicated that *S*_c_/*S* (*R*^2^ = 0.91, *P* < 0.05) and *T*_cw_ (*R*^2^ = 0.92, *P* < 0.01) had direct effects on *g*_m_ (Fig. 7). Variation of *S*_c_/*S* was mainly related to chloroplast number in mesophyll cells, although the variation of *S*_mes_/*S* may affect *S*_c_/*S*. Consistently, N form had a larger effect on *S*_c_/*S*_mes_ than *S*_mes_/*S* (Table 2). *T*_cw_ was related to cell wall compositions, and the relationship was mainly driven by the variations of lignin and hemicellulose. Notably, the path analysis did not show a direct influence of cell wall compositions on *g*_m_ (Fig. 7).

## Discussion

### The decline of leaf photosynthesis under NH_4_^+^ supplement is mainly related to decreased CO_2_ diffusion

In the present study, we found that NH_4_^+^ treatment had led to a substantial reduction in leaf *A* of *L. japonica* (Table 1). The declines of *g*_s_ and, in particularly, *g*_m_ are the major causes for the depressed leaf *A*, although *V*_cmax_ and *J*_max_ are also significantly reduced in NH_4_^+^ treatment (Table 1). The dominating effect of CO_2_ diffusion capacity in responses of photosynthesis to N form has also been found in previous studies [16, 19, 20]. The mechanisms relating to the influence of N form on *g*_m_ will be discussed later.

Here, a lower *g*_s_ in NH_4_^+^ supply was observed when comparing with the other two N forms (Table 1). The inhibition of *g*_s_ by NH_4_^+^ nutrition has been commonly reported in previous studies [35, 36], the lowered *g*_s_ under NH_4_^+^ supply is suggested to be a strategy to reduce NH_4_^+^ transportation into leaves [35]. Generally, *g*_s_ is regulated by stomatal anatomical features, and the reduction of stomatal aperture and stomatal number per unit leaf area would negatively affect *g*_s_ [37]. The impression of leaf indicates that *L. japonica* absorbs water and CO_2_ across hypostomatous leaf (Fig. S5, see online supplementary material). When the three treatments were compared, NO_3_^−^ supplement had a comparable stomatal density with NH_4_^+^, while the stomatal length and width were significantly higher than that of NH_4_^+^ supplement (Table S3, see online supplementary material). Guo *et al*. [38] reported that the *Arabidopsis* mutant with a low-level expression of nitrate transporter gene AtNRT1.1 possessed a lower *g*_s_, indicating that nitrate may play a role in stomatal opening. However, further research is necessary to explore the mechanisms underlying the regulation of N form on stomatal aperture.

Leaf biochemical functions, namely *V*_cmax_ and *J*_max_, are determined by Rubisco content and activity and are associated with both the N content and N allocation of leaves [24]. In the present study, the decreased biochemical functions in NH_4_^+^ supply were accompanied by a higher leaf N concentration per leaf area (*N*_a_) as well as the Rubisco content (Table S4, see online supplementary material); similar results were observed in Cao *et al*. [39]. Compared with NH_4_^+^treatment, the NO_3_^−^ and mixed N treatment had higher Rubisco activity, which might account for the increased *V*_cmax_ and *J*_max_ (Table 1; Table S4, see online supplementary material). Why NO_3_^−^ supplement would increase Rubisco activity of *L. japonica* remains unclear, however. One possible explanation may be the chloroplast *C*_c_ accumulation in a condition of NO_3_^−^ promoted the activation of Rubisco [40] (Table 1).

Moreover, the distribution of leaf N is crucial in determining *A*, by affecting N investment on photosynthetic apparatus (e.g., Rubisco enzyme) or non- photosynthetic apparatus (e.g., cell wall) [41, 42]. In comparison with NO_3_^−^ and mixed N treatments, NH_4_^+^ supplied plants possessed a lower photosynthetic N use efficiency (PNUE) (Table S4, see online supplementary material). According to Xue *et al*. [43], most of the PNUE variation could be explained by Rubisco N allocation fraction and *g*_m_. The influence of N form on N allocation has been reported in *Leymus chinensis* [44], and this differential leaf N allocation between N form may serve as a plant strategy to acclimate NH_4_^+^ stimuli.

### *g*_m_ reduction under NH_4_^+^ supplement is mainly related to declined *S*_c_/*S* and thicker cell walls

It has been frequently found that both *S*_c_/*S* and cell wall thickness serve as the main leaf anatomical factors that determine the largest *g*_m_ [10–12]. The results obtained from path analysis also suggested that the lower *g*_m_ in NH_4_^+^ treatment was mainly caused by the declined *S*_c_/*S* and the thicker cell walls (Fig. 7), although the lower *f*_ias_ in NH_4_^+^ treatment may restrain gas-phase CO_2_ diffusion (Table 2, Figs 4 and 5).

The value of *S*_c_/*S* is related to the arrangements of both mesophyll cells and chloroplasts (Fig. 5). Considering the little difference of *S*_mes_/*S* among three N forms, the decreased *S*_c_/*S* in NH_4_^+^ treatment was mainly related to its lower *S*_c_/*S*_mes_, which was in turn related to less chloroplasts in mesophyll cells in NH_4_^+^ treatment (Table 2 and Fig. 5; Fig. S2, see online supplementary material). Indeed, chloroplast development can be significantly affected by N form. Findings by Ariga *et al*. [45] revealed that *Arabidopsis* mutants having the lower expression of HOMEOBOX PROTEIN (a transcription factor depending on nitrate supply) possessed fewer chloroplasts in mesophyll cells. Similarly, An *et al*. [46] showed that overexpression of *PdGNC*, which is a nitrate-dependent GATA transcription factor, can lead to a significantly increased chloroplast number and photosynthesis in *Arabidopsis*. Therefore, N form can affect *g*_m_ and leaf photosynthesis by regulating chloroplast development (Fig. 7; Fig. S2, see online supplementary material).

It has been suggested that a larger photorespiration rate can lead to a lower apparent *g*_m_ because of the photorespiratory CO_2_ release and refixation [47]. In the present study, the variations in photorespiration flux partially represented by Γ^*^ may influence the observed *g*_m_ (Table S5, see online supplementary material). The magnitude of this effect depended largely on the effective location of CO_2_ release from (photo)respiratory in some cases [48]; for instance, the smaller *D*_chl_ in NO_3_^−^ would force the (photo)respired CO_2_ to release through chloroplasts rather than mixed with CO_2_ coming from the intercellular space, and the later would enlarge the CO_2_ diffusion resistance from cytosol (*r*_cyt_) and chloroplast envelope (*r*_env_) (Table 2 and Fig. 5). Despite the possibility for a large impact of photorespiration on *g*_m_ being low, because we have observed a correlation between *g*_m_-anatomy and *g*_m_-Harley (Fig. S3, see online supplementary material), we cannot exclude this possibility.

Cell walls are major restraints for CO_2_ diffusion inside leaves (Figs 4 and 5). It is suggested that cell wall resistance contributes 25%–50% of the overall mesophyll resistance [12]. The variation of the contribution is related to the effective porosity of cell walls [22], which cannot be directly measured due to methodological limitations. In the present study, *T*_cw_ was significantly higher in NH_4_^+^ nutrition compared to NO_3_^−^ nutrition (Table 2), which is one of the reasons for the declined *g*_m_ and *A* in NH_4_^+^ nutrition (Figs 3 and 7). However, the influence of N form on *T*_cw_ was species dependent. Gao *et al*. [19] reported that *T*_cw_ was larger in NH_4_^+^ nutrition than that in NO_3_^−^ nutrition, which agrees with the results in the present study. Similarly, Liu *et al*. [16] found a larger *T*_cw_ in NH_4_^+^ nutrition in female poplar; in contrast, *T*_cw_ was lower in NH_4_^+^ nutrition in male poplar. The mechanisms relating to the influence of N form on *T*_cw_ are not known, but the difference in cell wall compositions among N form treatments may account for the differential *T*_cw_.

### The influence of N form on cell wall compositions

Plant cell walls are reported to be susceptible to environmental perturbations and can respond very quickly to better cope with these changes [27, 49]. Similarly, we found that N form can significantly affect cell wall compositions in the present study. Generally, cellulose content was increased while hemicellulose and lignin were reduced in NO_3_^−^ nutrition (Fig. 6). The influence of N form on cell wall compositions may relate to the changing activity of critical enzymes. Landi and Esposito [29] found the co-expression of NO_3_^−^ transport and cellulose synthesis genes in *Arabidopsis*, which suggested a connection between nitrate assimilation and cellulose synthesis. In contrast, it has been found that nitrate leads to the down-regulation of a majority of genes involved in phenylpropanoid pathways, which will in turn result in the decrease of lignin content [30, 31]. Consistent findings reported by Głazowska *et al*. [31] indicated that the absence of a difference in lignin content among different N forms on early stage could be related to less tissue NO_3_^−^ accumulation in the mixed N and NO_3_^−^ treatments (Fig. 6). Furthermore, the pectin content was independent from N forms; the different responses patterns of pectin are assumed to be species dependent (e.g., succulent plants, [27]) or environment dependent [26].

The studies relating to the correlation between cell wall compositions and *g*_m_ are amounting, but the results are inconsistent. For instance, the multi-species analysis showed the variations in hemicelluloses and cellulose were negatively related with *g*_m_ [49]; other studies demonstrated a key role for pectin in determining *g*_m_ [21, 28]. The direct influence of cell wall compositions on *g*_m_ needs to be further studied. Most importantly, results of the recent studies may indicate the coordinated modifications in the composition and the thickness of cell wall in setting *g*_m_ [28, 29]. Similarly, the path analysis did support the influence of cell wall compositions on *g*_m_ by changing *T*_cw_ (Fig. 7). According to Carriquí *et al*. [49], the opposite relationship involving different phytogroups between *T*_cw_ and the absolute abundance of hemicellulose was identified. Hemicellulose is a heterogeneous polysaccharide, which plays a crucial role in strengthening cell walls by connecting cellulose microfibrils and attaches them to lignin [50]. An *in situ* study of wood structure revealed a positive relationship between the dynamic dissolution of lignin and hemicellulose and the *T*_cw_ reduction rate, which would facilitate better transportation of active compounds deep into the cell wall [51]. The decrease in the lignin and hemicellulose contents of the cell wall may thus be a key aspect involved in promoting CO_2_ diffusion in NO_3_^−^ supplement, by reducing *T*_cw_ (Fig. 6 and 7; Fig. S4, see online supplementary material).

## Conclusion

In summary, leaf photosynthesis of *L. japonica* was decreased in NH_4_^+^-fed leaves more than that in NO_3_^−^ nutrition. Limitation analysis revealed that the variation of *g*_m_ explained most of the reduction of photosynthesis under NH_4_^+^ treatment, followed by *g*_s_ and biochemical capacity. Variations of *g*_m_ under different N forms primarily correlated with changes in leaf anatomical traits of *T*_cw_ and chloroplast numbers, and cell wall was thicker and chloroplast number was lower in NH_4_^+^-fed leaves compared to NO_3_^−^ nutrition. Furthermore, cell wall compositions were substantially affected by N form, and lignin and hemicellulose were the pivotal cell wall components in regulating *g*_m_ among N form. Compared to NO_3_^−^, the decreased *g*_s_ in NH_4_^+^ condition was mainly mediated by the decrement of stomatal aperture, while the reduction of biochemical capacity in NH_4_^+^ is primarily associated with the differences in nitrogen allocation and the decrease of Rubisco activity. This research emphasized the significance of leaf anatomical changes in regulating *g*_m_ and leaf photosynthesis among different N forms, and further research is required to explore the mechanisms underlying the association between N assimilation and the synthesis of cell walls.

## Materials and methods

### Plant material and treatment

The experiments were carried out in the intelligent greenhouse of Nanjing Agricultural University, China. Seedlings of *L. japonica* were maintained in the greenhouse with a day/night temperature of 28°C/18°C. Light intensity was maintained at 1000 μmol (photons) m^−2^ s^−1^ at the leaf level with SON-T AGRO 400 W bulbs, and the photoperiod was set to 14 h light/10 h dark. Before treatment, seedlings were supplied with a half-strength nutrient solution for 2 weeks and full-strength nutrient solution for another 2 weeks for adaption (for nutrient solution composition, see Table S6, see online supplementary material). Homogeneous seedlings were then selected for the N form treatment. The provided N concentration was 2.8 mM, and it was supplied in three distinct N forms: (NH_4_)_2_SO_4_ alone, Ca(NO_3_)_2_ alone, and a combination of 50% (NH_4_)_2_SO_4_ and 50% Ca(NO_3_)_2_. CaCl_2_ was added to the NH_4_^+^ and mixed treatments to adjust Ca concentration to the level of the NO_3_^−^ treatment (2.8 mM).

### Gas exchange and chlorophyll fluorescence measurement

Four weeks after treatments started, leaf gas exchange and chlorophyll fluorescence of new fully-expanded leaves were simultaneously measured with a LI-6800 portable photosynthetic analyser equipped with a 2 cm^2^ fluorometer chamber. The leaf temperature with the chamber was 25 ± 0.5°C, the relative humidity was maintained at 40–60%, air flow rate was 500 μmol s^−1^, CO_2_ concentration (*C*_a_) was 400 ± 6 μmol mol^−1^, and the photosynthetic photon flux density (PPFD) was set as 1000 μmol photons m^−2^ s^−1^, and the light quality was set to10/90 of blue/red light. When gas exchange had stabilized for at least 20 min, *A*, *g*_s_, *C*_i_, steady-state fluorescence yield (*F*_s_), and the maximum fluorescence (*F*_m_′) were measured simultaneously with a multiphase flash. The effective quantum efficiency of photosystem II (Φ_PSII_) was quantified as Φ_PSII_ = (*F*_m_′–*F*_s_)/*F*_m_′. Five individual replicates were conducted for each treatment.

For photosynthetic CO_2_ responses (*A*/*C*_i_) measurement, the same environmental conditions within the leaf chamber were controlled. After attaching the leaves to the leaf chamber for 20 min, *C*_a_ was set in a descending series of 400, 350, 300, 250, 200, 150, 100, 400, 450, 500, 550, 650, 800, and 1000 μmol CO_2_ mol^−1^. Each CO_2_ concentration was allowed to equilibrate for approximately 2–3 min. The *V*_cmax_ and *J*_max_ were calculated using Sharkey *et al*.’s [52] model. CO_2_ leakage through the gaskets was inevitable in gas exchange measurement [53]. To reduce the effect of leakage, leaks were checked when the CO_2_ concentration within the leaf chamber was 800 and 1000 μmol CO_2_ mol^−1^, to ensure no more than 0.2 μmol CO_2_ mol^−1^ leaked. When stable photosynthetic conditions were reached, the data were recorded after CO_2_ concentration and water vapour between the leaf and the reference chamber were automatically matched [54].

### Estimation of *g*_m_ using the variable *J* method

Both *g*_m_ and *C*_c_ were estimated according to Harley *et al*. [55]:

(1) \begin{equation*} {g}_{\mathrm{m}}=\frac{A}{C_{\mathrm{i}}-\frac{\Gamma^{\ast}\left(J+8\left(A+{R}_{\mathrm{d}}\right)\right)}{J-4\left(A+{R}_{\mathrm{d}}\right)}} \end{equation*}

(2) \begin{equation*} {\mathrm{C}}_{\mathrm{c}}={\mathrm{C}}_{\mathrm{i}}-\frac{\mathrm{A}}{{\mathrm{g}}_{\mathrm{m}}} \end{equation*}

where the photosynthetic electron transport rate (*J*) was calculated as *J* = Φ_PSII_ × PPFD × α × β. In this context, α represents the absorption of light by leaf, while β denotes the fraction of quanta absorbed by PSII. The product of α × β was estimated according to Valentini *et al*. [56], from the relationship between Φ_PSII_ and Φ_CO2_ (quantum efficiency of CO_2_ fixation). Because no substantial variation in α × β was observed across the three treatments (Table S5, see online supplementary material), the average α × β value of 0.42 was used to calculate *g*_m_ in all three treatments.

The apparent intercellular CO_2_ compensation point of net CO_2_ assimilation rate in the absence of respiration (*C*_i_^*^) and day respiration rate (*R*_d_) were measured according to the method proposed by Brooks and Farquhar [57]. The methods relied on measuring the *A*/*C*_i_ relationship at three different PPFDs (150, 300, and 600 μmol photons m^−2^ s^−1^) with each having four ambient CO_2_ concentration of 25, 50, 75, and 100 μmol CO_2_ mol^−1^. Prior to initiating measurements, leaves were placed in a condition at a PPFD of 600 μmol photons m^−2^ s^−1^ and a CO_2_ concentration of 100 μmol CO_2_ mol^−1^ for 30 min for adaption. Their linear regressions were then predicted to converge at one point where the x-axis and y-axis of the point were defined as *C*_i_^*^ and *R*_d_. The CO_2_ compensation point in the absence of mitochondrial respiration (Γ^*^) was then calculated as follows:


(3) \begin{equation*} {\Gamma}^{\ast }={C}_{\mathrm{i}}^{\ast }+\frac{R_{\mathrm{d}}}{g_{\mathrm{m}}} \end{equation*}

Values of Γ^*^, *C*_i_^*^, and *R*_d_ in three treatments could be found in Table S5 (see online supplementary material).

### Leaf area, leaf N content, and Ribulose-1,5-bisphosphate carboxylase/oxygenase (rubisco) content and activity

New fully-expanded leaf was randomly selected and photographed. The *L*_A_ obtained from each image was calculated using Image-Pro Plus. Subsequently, leaves were dried at 105°C for 15 min, and at 65°C to reach a stable weight and samples were weighed. Leaf mass area (*M*_A_) was calculated as: *M*_A_ = leaf dry weight/*L*_A_. To determine per leaf area concentration of N (*N*_a_), dried leaf samples were digested with H_2_SO_4_-H_2_O_2_ at 280°C, and an Auto–analyzer 3 digital colorimeter (AA3, Bran+Luebbe Inc., Norderstedt, Germany) was used to determine the N concentration according to the methods of Guo *et al*. [58].

Content and activity of Rubisco were determined by Plant Rubisco Elisa Kit (Jiangsu Enzyme Free Biotechnology Co., Ltd, Nanjing, China). For each replication, 0.10 g leaf sample was grinded to homogenate in a pre-cooled mortar, with 1.0 mL chilled PBS solution (pH 7.2–7.4). The mixture was centrifuged at 8000 × *g* for 20 min and the supernatant was used for the determination following the manufacturer’s protocol.

### Quantitative limitation analysis of *a*

The relative limitations on leaf photosynthesis resulting from *g*_s_ (*l*_s_), *g*_m_ (*l*_m_), and biochemical capacity (*l*_b_), where the values of *l*_s_, *l*_m_, and *l*_b_ added up to 100%. According to Grass and Magnani [59]:

(4) \begin{equation*} {\mathrm{l}}_{\mathrm{s}}=\frac{{\mathrm{g}}_{\mathrm{tot}}/{\mathrm{g}}_{\mathrm{s}\mathrm{c}}\ast \mathrm{\partial A}/\partial{\mathrm{C}}_{\mathrm{C}}}{{\mathrm{g}}_{\mathrm{tot}}+\mathrm{\partial A}/\partial{\mathrm{C}}_{\mathrm{C}}} \end{equation*}

(5) \begin{equation*} {\mathrm{l}}_{\mathrm{m}}=\frac{{\mathrm{g}}_{\mathrm{tot}}/{\mathrm{g}}_{\mathrm{m}}\ast \mathrm{\partial A}/\partial{\mathrm{C}}_{\mathrm{C}}}{{\mathrm{g}}_{\mathrm{tot}}+\mathrm{\partial A}/\partial{\mathrm{C}}_{\mathrm{C}}} \end{equation*}

(6) \begin{equation*} {\mathrm{l}}_{\mathrm{b}}=\frac{{\mathrm{g}}_{\mathrm{tot}}}{{\mathrm{g}}_{\mathrm{tot}}+\mathrm{\partial A}/\partial{\mathrm{C}}_{\mathrm{C}}} \end{equation*}

where *g*_tot_ represents the CO_2_ conductance from the leaf surface to carboxylation sites inside the chloroplast, which can be determined through *g*_sc_ and *g*_m_. ∂*A*/ ∂*C*_c_ was estimated according to the slope of the *A*/*C*_c_ curves over a *C*_c_ range of 50–100 μmol mol^−1^.

### Anatomical analysis

After the gas exchange measurements, 5 × 5 mm leaf sections were collected between the main veins from the same leaves for anatomical measurements. The preparation of paraffin and resin sections followed the method of Lu *et al*. [14] and Gao *et al*. [19]. The safranin-fast green staining was applied to the paraffin sections, which were then observed using a Nikon Eclipse ci microscope equipped with a Nikon digital microscope camera. The images were captured at a 400× magnification to measure the *T*_L_, *T*_mes_, with ≥20 fields of view per treatment. Ultrathin resin sections were contrasted with 2% uranyl acetate and lead citrate, then viewed with transmission electron microscopy (TEM). Graphs were acquired at a direct magnification of 2000–8000× to measure *T*_cw_, *L*_chl_ and *T*_chl_, *D*_chl-chl_, *L*_cyt_, and chloroplast numbers per mesophyll cell, with ≥30 fields micro-pictures for each treatment. All the images were analysed using Image-Pro Plus software.

The leaf density (*D*_L_) and the *f*_ias_ was calculated using measurements from light-microscope graphs. *D*_L_ and *f*_ias_ were determined as follows:

(7) \begin{equation*} {D}_{\mathrm{L}}=\frac{M_{\mathrm{A}}}{T_{\mathrm{L}}} \end{equation*}

(8) \begin{equation*} {\mathrm{f}}_{\mathrm{ias}}=1-\frac{\Sigma{\mathrm{S}}_{\mathrm{S}}}{{\mathrm{T}}_{\mathrm{mes}}\ \mathrm{W}} \end{equation*}

where *M*_A_ represents the weight of specific leaf (mg cm^−2^), Σ*S*_s_ denotes the combined cross-section area of mesophyll cells, and *W* corresponds to the width of the measured framed range.

*S*_c_/*S* and *S*_mes_/*S* were calculated according to the method of Evans *et al*. [60] and Syvertsen *et al*. [61].

(9) \begin{equation*} {\mathrm{S}}_{\mathrm{mes}}/\mathrm{S}=\frac{{\mathrm{L}}_{\mathrm{mes}}}{\mathrm{W}}\ast \mathrm{F} \end{equation*}

(10) \begin{equation*} {\mathrm{S}}_{\mathrm{c}}/\mathrm{S}=\frac{{\mathrm{L}}_{\mathrm{c}}}{\mathrm{W}}\ast \mathrm{F}=\frac{{\mathrm{L}}_{\mathrm{c}}}{{\mathrm{L}}_{\mathrm{mes}}}\ast{\mathrm{S}}_{\mathrm{mes}}/\mathrm{S} \end{equation*}

where the *L*_mes_ is the total mesophyll surface length exposed to the intercellular air space; *L*_c_ represents the chloroplast surface length facing the intercellular air space. The curvature correction factor *F* was calculated using the method of Thain [62], depending on the shape of the mesophyll cell. Briefly, based on differences between palisade and spongy cell, we derived *F* as a weighted average from both spongy (*F*_1_) and palisade (*F*_2_) mesophyll distributions:

(11) \begin{equation*} {\mathrm{F}}_1=\frac{1+2{\overline{\mathrm{b}}}_1/{\overline{\mathrm{a}}}_1}{1+4{\overline{\mathrm{b}}}_1/\mathrm{\pi} {\overline{\mathrm{a}}}_1} \end{equation*}

(12) \begin{equation*} {F}_2=\left[\left(\overline{a}/\overline{b}\right)+\left(1/e\right)\ {\sin}^{-1}e\right]/E \end{equation*}

where $\overline{a}$ and $\overline{b}$ represent the mean mesophyll length and mean mesophyll thickness. The eccentricity *e* and elliptical integral *E* were calculated as proposed by Weast [63]:


(13) \begin{equation*} \mathrm{e}=\sqrt{1-{\left(\overline{\mathrm{a}}\right)}^2/{\left(\overline{\mathrm{b}}\right)}^2} \end{equation*}

(14) \begin{equation*} E\approx\frac{\mathrm{\pi}}{2}\ \left(1-\frac{e^2}{4}-\frac{3{e}^4}{64}-\frac{5{e}^3}{256}\right) \end{equation*}

For stomatal features measurement, the impression protocol was conducted for the assessment of leaf stomatal density and aperture (length and width) [64]. Images were captured with an optical microscope system (Olympus IX71, Olympus Optical, Japan) at a magnification of 200× and 400× to measure the leaf stomatal density and at a magnification of 1000× for the measurement of the length and width of stomatal aperture, with ≥20 fields of view per treatment.

### *g*_m_ modelled from anatomical characteristics

The one-dimensional gas diffusion model modified by Tomás *et al*. [34] was used to determine *g*_m_, which is divided into leaf gas and liquid components:

(15) \begin{equation*} {\mathrm{g}}_{\mathrm{m}}=\frac{1}{\frac{1}{{\mathrm{g}}_{\mathrm{ias}}}+\frac{\mathrm{R}{\mathrm{T}}_{\mathrm{k}}}{\mathrm{H}\ast{\mathrm{g}}_{\mathrm{liq}}}} \end{equation*}

where *g*_ias_ represents the gas-phase conductance from substomatal to the cell wall’s outer surface; *g*_liq_ denotes the liquid conductance from the cell wall’s outer surface to chloroplasts. *R* is the gas constant, *T*_k_ is the absolute temperature (K), and *H* represents Henry’s law constant (2943.3 Pa m^3^ K^−1^ mol^−1^ for CO_2_) [19].

In this model, the gas-phase conductance (*g*_ias_) depends on *f*_ias_ and effective diffusion path in the gas phase (∆*L*_ias_). *g*_ias_ was calculated as follows:

(16) \begin{equation*} {g}_{\mathrm{ias}}=\frac{1}{r_{\mathrm{ias}}}=\frac{D_{\mathrm{a}}\ast{f}_{\mathrm{ias}}}{\Delta{L}_{\mathrm{ias}}\ast \varsigma } \end{equation*}

where ς is the diffusion path tortuosity [19, 34] and *D*_a_ (m^2^ s^−1^) is the diffusion coefficient for CO_2_ in the gas-phase [34]. ∆*L*_ias_ is defined as half of *T*_mes_.

The *g*_liq_ was determined by various mesophyll cell properties, including conductance in cell walls (*g*_cw_), plasmalemma (*g*_pl_), cytosol (*g*_cyt_), stroma (*g*_st_), and chloroplast envelope (*g*_env_). Thus, *g*_liq_ was defined as:

(17) \begin{equation*} {g}_{\mathrm{liq}}=\frac{S_{\mathrm{c}}}{\left({r}_{\mathrm{c}\mathrm{w}}+{r}_{\mathrm{pl}}+{r}_{\mathrm{c}\mathrm{yt}}+{r}_{\mathrm{en}}+{r}_{\mathrm{st}}\right)S} \end{equation*}

where *r*_cw_, *r*_pl_, *r*_cyt_, *r*_en_, and *r*_st_ are the reciprocal terms of *g*_cw_, *g*_pl_, *g*_cyt_, *g*_env_, and *g*_st_, respectively. Alternatively, according to Tholen *et al*. [65], CO_2_ diffusion inside the cell differs, such that one route involves cell wall parts and chloroplasts (*g*_cel,1_), whereas the other route involves inter-chloroplast areas (*g*_cel,2_). The corresponding resistance was expressed as *r*_cel,1_ and *r*_cel,2_. As a result, the equation for *g*_liq_ was transformed into:


(18) \begin{equation*} {g}_{\mathrm{liq}}=\frac{S_{\mathrm{mes}}}{\left({r}_{\mathrm{cw}}+{r}_{\mathrm{pl}}+{r}_{\mathrm{cel},1}+{r}_{\mathrm{cel},2}\right)S} \end{equation*}

The liquid-phase CO_2_ diffusion pathway, whether for *g*_cw_, *g*_cyt_, or *g*_st_, is expressed as follows:

(19) \begin{equation*} {\mathrm{g}}_{\mathrm{i}}=\frac{1}{{\mathrm{r}}_{\mathrm{i}}}=\frac{{\mathrm{r}}_{\mathrm{f},\mathrm{i}}\ast{\mathrm{D}}_{\mathrm{w}}\ast{\mathrm{p}}_{\mathrm{i}}}{\Delta{\mathrm{L}}_{\mathrm{i}}} \end{equation*}

where *r*_f,i_ is a factor that accounts for the decreased CO_2_ diffusion in the aqueous phase vs. free diffusion in water; this factor was set to 1.0 for the cell wall and 0.3 for the cytosol and stroma. Δ*L*_i_ (m) represents the distance that CO_2_ diffuse in the corresponding component, while *p*_i_ (m^3^ m^−3^) reflects the effective porosity. Evidence showed that the effective porosity varied with cell wall thickness [53]. Therefore, a least squared iterative analysis was used by varying the *p*_i_ of the cell wall to obtain the best fit value (highest *R*^2^) between the measured and modelled *g*_m_ value [34]. As a result, *p*_i_ was taken as 0.30. *D*_w_ denotes the CO_2_ diffusion coefficient in the aqueous phase. Both *g*_pl_ and *g*_env_ were set as 0.0035 m s^−1^ [60].

### Quantitation of the anatomical limitation of *g*_m_

The factors influencing *g*_m_ consist of both the conductance in gas-phase (*l*_ias_) and in liquid-phase (*l*_i_). The *l*_ias_ can be calculated as:


(20) \begin{equation*} {\mathrm{l}}_{\mathrm{ias}}=\frac{{\mathrm{g}}_{\mathrm{m}}}{{\mathrm{g}}_{\mathrm{ias}}} \end{equation*}

The share of *g*_m_ contributed by the cellular-phase conductance (*l*_i_), an indicator of the limitation of the cell wall, cellular contents, as well as the plasmalemma, was determined as:

(21) \begin{equation*} {\mathrm{l}}_{\mathrm{i}}=\frac{{\mathrm{g}}_{\mathrm{m}}}{{\mathrm{g}}_{\mathrm{i}}\ast \frac{{\mathrm{S}}_{\mathrm{m}\mathrm{es}}}{\mathrm{S}}} \end{equation*}

where *g*_i_ represents the conductance for diffusion in each corresponding pathway.

### Cell wall compositions determination

Dynamic analyses of cell wall compositions were conducted using plant samples collected on four specific sampling days for each treatment: days 0 (before treatment), 7, 14, and 28. Leaves were collected at the early morning (05:30–06:00) to minimize leaf starch content and 1.0 g of each leaf sample was boiled in absolute ethanol until the tissue became bleached. Alcohol-soluble residues were eliminated with acetone, then the samples were air-dried and homogenized, which yielded the alcohol-insoluble residue (AIR). The AIR was identified as the crude cell wall material and was used for analyses of cellulose, hemicellulose, pectin, and lignin.

In the first step, 3 mg of each AIR were hydrolyzed using trifluoroacetic acid and subjected to 121°C for one hour. Subsequently, the mixture was centrifuged at 13000 × *g*. The supernatant containing non-cellulosic cell wall components was maintained at 4°C for the quantification of pectin and hemicelluloses; the pellet was used for the determination of cellulose quantification. The same protocol was used to calculate the cellulose and hemicellulose contents via the phenol sulfuric acid method [66]. For pectin quantification, a galacturonic acid calibration curve was carried out in accordance with Blumenkrantz and Asboe-Hansen [67]. Lignin was quantified using 20 mg of AIR sample, which was processed via the acetyl bromide method [68]. The spectrophotometer used for measurements was HBS-1096A (Shanghai, China).

### Statistical analysis

Data analyses were conducted using SPSS 25.0 to perform one-way ANOVA. To determine significant differences between treatments, the least significant difference (LSD) test was employed at a significance level of *P* < 0.05. Graphical depiction and regression analyses were carried out using Origin Pro 2021. PLS-PM analysis was performed with the *R* package ‘plspm’ using *R* statistical software (v.4.0.2).

## Acknowledgements

This work was financially supported by the National Natural Science Foundation of China (32072673), the Fundamental Research Funds for the Central Universities (KYGD202007), the Young Elite Scientists Sponsorship Program by CAST (2018QNRC001), and the Innovative Research Team Development Plan of the Ministry of Education of China (IRT_17R56).

## Author contributions

S.G. and Y.C. conceived the idea and designed the experiment; Y.C., Y.P., Y.Y., and T.L. completed the experiments; Y.C. analysed the data and wrote the manuscript; Y.L. and Y.P. helped in manuscript revising; S.G. and M.W. provided funding support. All the authors contributed critically to the drafts and gave final approval for publication.

## Data availability statement

The data underlying this article are available in the article and in its online supplementary material.

## Conflict of interests

The authors declare that the research was conducted in the absence of any commercial or financial relationships that could be construed as a potential conflict of interest.

## Supplementary information

Supplementary data is available at *Horticulture Research* online.

## Supplementary Material

Web_Material_uhae112
