# Supplementary material for: Variation of mesophyll conductance mediated by nitrogen form is related to changes in cell wall property and chloroplast number
Source: Hortic Res. 2024 Feb 22;11(6):uhae112. doi: 10.1093/hr/uhae112 (PMC11197310; doi:10.1093/hr/uhae112)
Supplement: Web_Material_uhae112 [file web_material_uhae112.docx]

**Supplementary data**

**Title:** Variation of mesophyll conductance mediated by nitrogen form is related to changes in cell wall property and chloroplast number

**Authors:** Yiwen Cao, Yonghui Pan, Yating Yang, Tianheng Liu, Min Wang, Yong Li*, Shiwei Guo*

**Table S1** Effect of different nitrogen (N) forms on integral leaf variables.

**Table S2** Effect of different N forms on chloroplast morphology characteristics.

**Table S3** Effect of different N forms on the stomatal density and aperture.

**Table S4** Effect of different N forms on leaf N content per leaf area (*N*_a_), the photosynthetic N use efficiency (PNUE), and Ribulose-1,5-bisphosphate carboxylase/oxygenase (Rubisco) traits.

**Table S5** Parameters in relation to the calculation of mesophyll conductance (*g*_m_) by gas exchange and chlorophyll fluorescence.

**Table S6** Nutrient composition of different N form treatment. The pH of nutrient solution was adjusted to 6.0.

**Fig. S1** Correlations of leaf gas exchange parameters.

**Fig. S2** Correlations of chloroplast surface exposed to intercellular air spaces per leaf area (*S*_c_/*S*) and chloroplast numbers in spongy parenchyma (*N*_chl1_) **(a)**, chloroplast numbers in palisade cell (*N*_chl2_) **(b)**, and the correlations of chloroplast surface exposed to mesophyll cell surface area per leaf area (*S*_c_/*S*_mes_) and *N*_chl1_ **(c)**, *N*c_hl2_ **(d)**.

**Fig. S3** Correlations of *g*_m_ estimated from *g*_m_-Harley and *g*_m_-anatomy*.*

**Fig. S4** Correlations of *g*_m_ and different cell wall components (data obtained from day 28).

**Fig. S5** Effect of different N forms on leaf stomatal microscope characteristics of *Lonicera japonica*.

**Table S1** Effect of different nitrogen (N) forms on integral leaf variables.

| Treatment | *L*_A_  (cm^2^) | *M*_A_  (mg cm^-2^) | *T*_L_  (μm) | *T*_mes_  (μm) |
| --- | --- | --- | --- | --- |
| A | 8.4±0.7b | 4.5±0.3a | 115±5a | 83.5±7.5a |
| AN | 13.8±1.50a | 4.8±0.4a | 107±5a | 83.5±4.1a |
| N | 13.3±0.8a | 4.0±0.3a | 110±4a | 86.3±4.4a |

*L*_A_, leaf area; *M*_A_, leaf mass per area; *T*_L_, leaf thickness; *T*_mes_, mesophyll thickness. A, AN, and N represent N form of NH_4_^+^ alone, a mixed N supply, and NO_3_^–^ alone, respectively. Data are means ± SE (n=4). The data followed by different letters are significant at *P* < 0.05 level.

**Table S2** Effect of different N forms on chloroplast morphology characteristics.

| Treatment | *L*_chl_  (μm) | *T*_chl_  (μm) |
| --- | --- | --- |
| A | 6.2±0.5a | 2.0±0.1a |
| AN | 5.6±0.2a | 2.0±0.1a |
| N | 5.6±0.4a | 1.8±0.1a |

*L*_chl_, chloroplast length; *T*_chl_, chloroplast thickness. A, AN, and N represent N form of NH_4_^+^ alone, a mixed N supply, and NO_3_^–^ alone, respectively. Data are means ± SE (n=4). The data followed by different letters are significant at *P* < 0.05 level.

| Treatment | *D*_aba_  (No. mm^-2^) | *L*_aba_  (μm) | *W*_aba_  (μm) | *L*_aba_/*W*_aba_ |
| --- | --- | --- | --- | --- |
| A | 602±31b | 10.82±0.53b | 4.84±0.52b | 2.28±0.21a |
| AN | 869±41a | 11.49±1.17b | 5.08±0.77ab | 2.31±0.19a |
| N | 622±27b | 13.40±0.55a | 5.81±0.53a | 2.35±0.20a |

**Table S3** Effect of different N forms on the stomatal density and aperture.

Note：*D*_aba_, stomatal density at adaxial leaf surface; *L*_aba_, length of stomatal aperture at adaxial leaf surface; *W*_aba_, width of stomatal aperture at adaxial leaf surface; *L*_aba_/*W*_aba_, ratio of *L*_aba_/*W*_aba_. The data followed by different letters are significant at *P* < 0.05 level.

**Table S4** Effect of different N forms on leaf N content per leaf area (*N*_a_), the photosynthetic N use efficiency (PNUE), and Ribulose-1,5-bisphosphate carboxylase/oxygenase (Rubisco) traits.

| Treatment | *N*_a_ (g m^-2^) | PNUE | Activity of Rubisco (U L^-1^) | Content of Rubisco (mg g^-1^) |
| --- | --- | --- | --- | --- |
| A | 1.5±0.1a | 9.5±1.0c | 91.9±5.4b | 98.2±6.0a |
| AN | 1.4±0.1a | 13.2±0.8b | 119.0±14.1a | 98.3±6.2a |
| N | 1.2±0.1b | 16.9±0.9a | 129.4±9.3a | 87.9±7.7b |

PNUE=net photosynthesis rate/*N*_a_. A, AN, and N represent N form of NH_4_^+^ alone, a mixed N supply, and NO_3_^–^ alone, respectively. Data are means ± SE (n=5). The data followed by different letters are significant at *P* < 0.05 level.

**Table S5** Parameters in relation to the calculation of mesophyll conductance (*g*_m_) by gas exchange and chlorophyll fluorescence.

| Treatment | α×β | *J* (µmol m^-2^ s^-1^) | *R*_d_ (μmol m^-2^ s^-1^) | *C*_i_^*^(μmol mol^-1^) | Γ^*^(μbar) |
| --- | --- | --- | --- | --- | --- |
| A | 0.41±0.01a | 121.5±1.7c | 0.70±0.03a | 48.9±0.2a | 41.0±0.4b |
| AN | 0.43±0.01a | 136.8±2.7b | 0.59±0.02b | 48.6±0.1a | 44.1±0.2a |
| N | 0.42±0.01a | 154.8±0.9a | 0.59±0.03b | 48.8±0.3a | 43.7±0.3a |

α is the leaf absorption and β is the proportion of quanta absorbed by PSII; *J*, electron transfer rate; *R*_d_, day respiration rate; Γ^*^, the CO_2_ compensation point in the absence of mitochondrial respiration. A, AN, and N represent N form of NH_4_^+^ alone, a mixed N supply, and NO_3_^–^ alone, respectively. Data are means ± SE (n=5). The data followed by different letters are significant at *P* < 0.05 level.

**Table S6** Nutrient composition of different N form treatment.

Note: Dicyandiamide was added to each nutrition solution as a nitrification inhibitor, and the nutrition solution was aerated for 1 h/1 h day/night and was renewed every 4 days, whereas the pH was adjusted daily to 6.0 ± 0.1 with 1 M HCl and 1 M NaOH.

| Compounds | Concentration | A | AN | N |
| --- | --- | --- | --- | --- |
| (NH_4_)_2_SO_4_ | mmol L^-1^ | 2.86 | 1.43 | - |
| Ca (NO_3_)_2_·4H_2_O | mmol L^-1^ | - | 1.43 | 2.86 |
| CaCl_2_·2H_2_O | mmol L^-1^ | 2.86 | 1.43 | - |
| MgSO_4_·7H_2_O | mmol L^-1^ | 2.00 | 2.00 | 2.00 |
| K_2_SO_4_ | mmol L^-1^ | 1.03 | 1.03 | 1.03 |
| KH_2_PO_4_ | mmol L^-1^ | 0.32 | 0.32 | 0.32 |
| MnCl_2_·4H_2_O | mmol L^-1^ | 9.10×10^−3^ | 9.10×10^−3^ | 9.10×10^−3^ |
| (NH_4_)_6_Mo_7_O_24_·4H_2_O | mmol L^-1^ | 5.20×10^−4^ | 5.20×10^−4^ | 5.20×10^−4^ |
| H_3_BO_3_ | mmol L^-1^ | 3.70×10^−2^ | 3.70×10^−2^ | 3.70×10^−2^ |
| ZnSO_4_·7H_2_O | mmol L^-1^ | 1.50×10^−4^ | 1.50×10^−4^ | 1.50×10^−4^ |
| CuSO_4_·5H_2_O | mmol L^-1^ | 1.60×10^−4^ | 1.60×10^−4^ | 1.60×10^−4^ |
| Fe–EDTA | mmol L^-1^ | 3.58×10^−2^ | 3.58×10^−2^ | 3.58×10^−2^ |
| NaSiO_3_·9H_2_O | mmol L^-1^ | 1.00×10^−4^ | 1.00×10^−4^ | 1.00×10^−4^ |
| Dicyandiamide | ‰ | 1.00 | 1.00 | 1.00 |


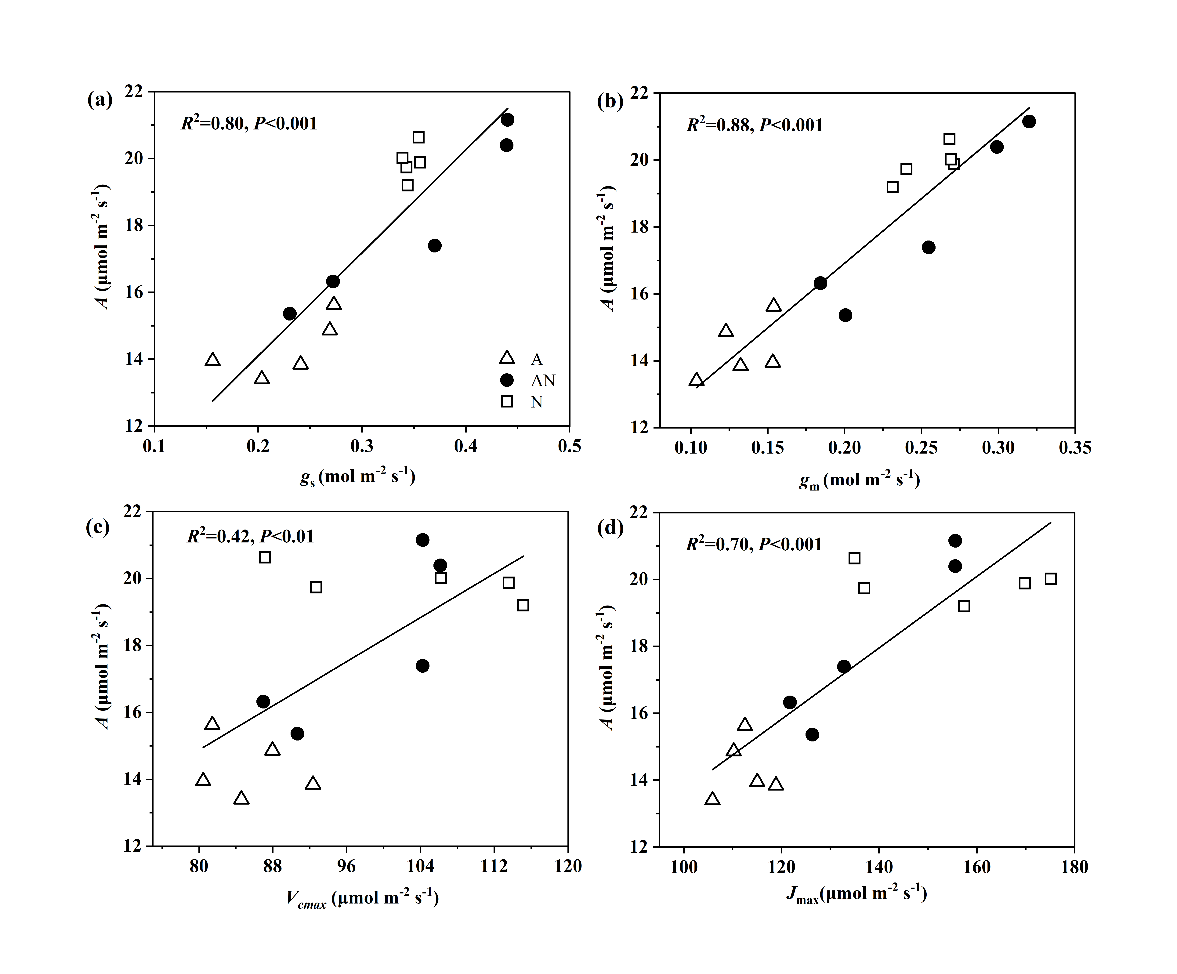


**Fig.** **S1** Correlations of leaf gas exchange parameters. *A*, net photosynthetic rate; *g*_s_, stomatal conductance; *V*_cmax_, the maximum carboxylation rate, *J*_max_, the maximum electron transfer rate. A, AN, and N represent N form of NH_4_^+^ alone (open triangles), a mixed N supply (closed circles), and NO_3_^–^ alone (open squares), respectively. Data are fitted by linear regression (n=5).


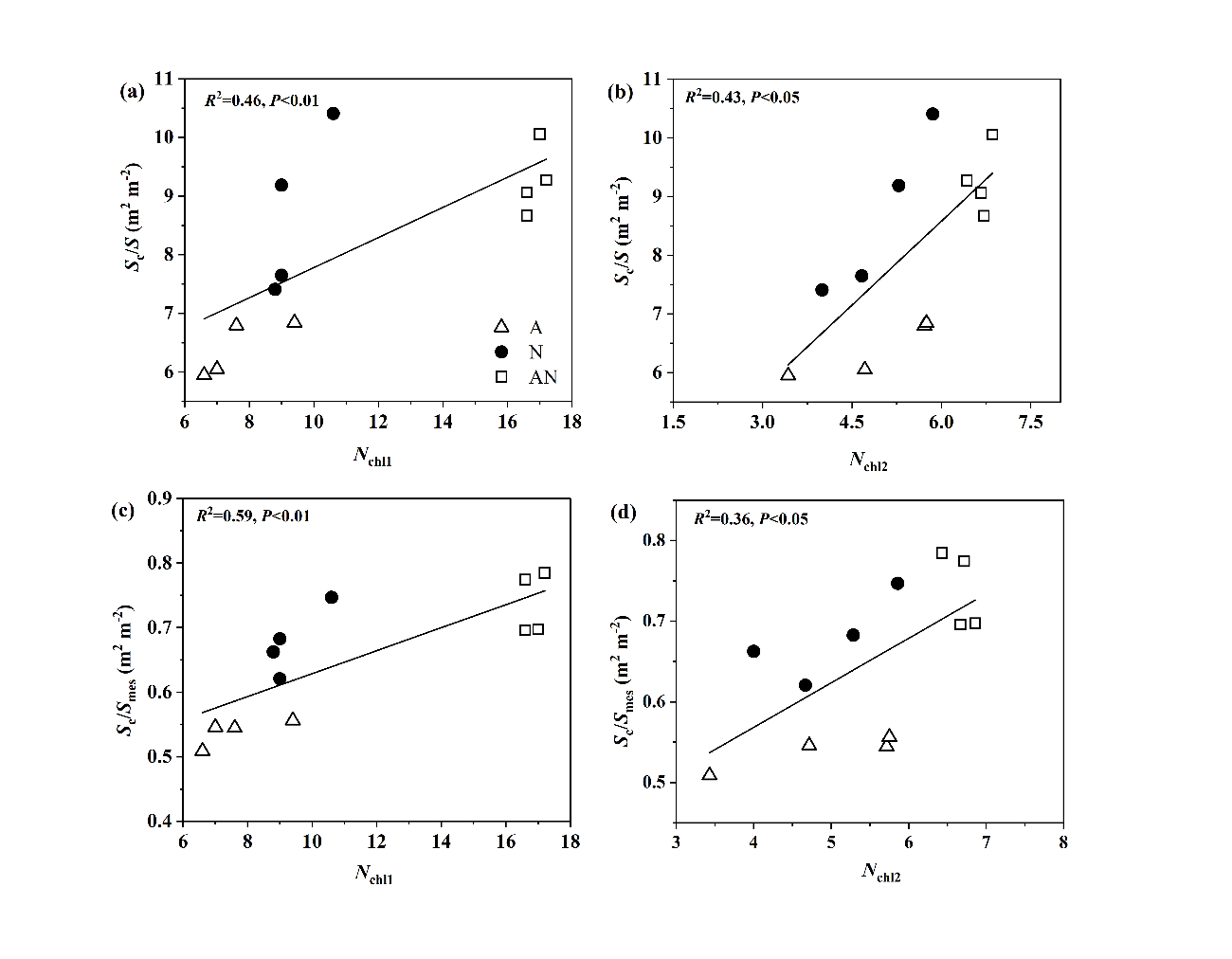


**Fig. S2** Correlations of chloroplast surface exposed to intercellular air spaces per leaf area (*S*_c_/*S*) and chloroplast numbers in spongy parenchyma (*N*_chl1_) **(a)**, chloroplast numbers in palisade cell (*N*_chl2_) **(b)**, and the correlations of chloroplast surface exposed to mesophyll cell surface area per leaf area (*S*_c_/*S*_mes_) and *N*_chl1_ **(c)**, *N*c_hl2_ **(d)**. A, AN, and N represent treatments with NH_4_^+^ alone (open triangles), a mixed N supply (closed circles), and NO_3_^–^ alone (open squares), respectively. Data are fitted by linear regression (n=4).


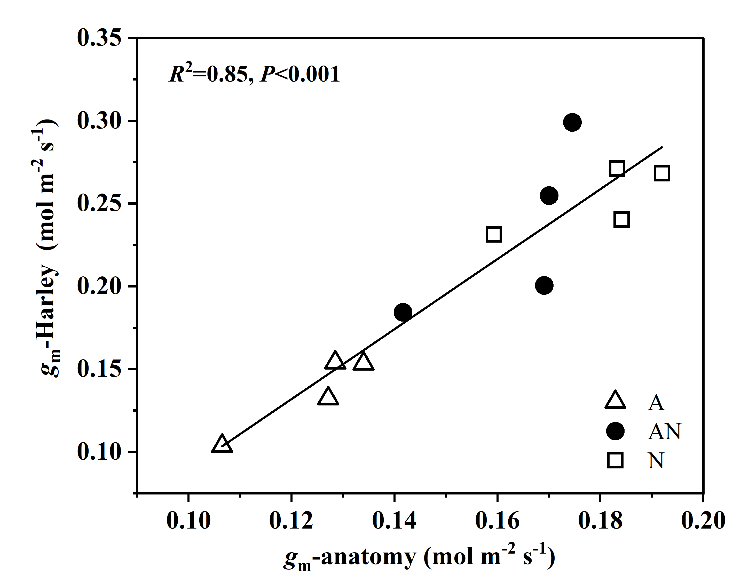


**Fig. S3** Correlations of *g*_m_ estimated from *g*_m_-Harley and *g*_m_-anatomy. *g*_m_-Harley, a *g*_m_ values that were calculated using the variable *J* method (Harley *et al*. 1992); *g*_m_-anatomy, a *g*_m_ values that were calculated using the one-dimensional gas diffusion model (Tomás *et al*. 2013). A, AN, and N represent N form of NH_4_^+^ alone (open triangles), a mixed N supply (closed circles), and NO_3_^–^ alone (open squares), respectively. Data are fitted by linear regression (n=4).


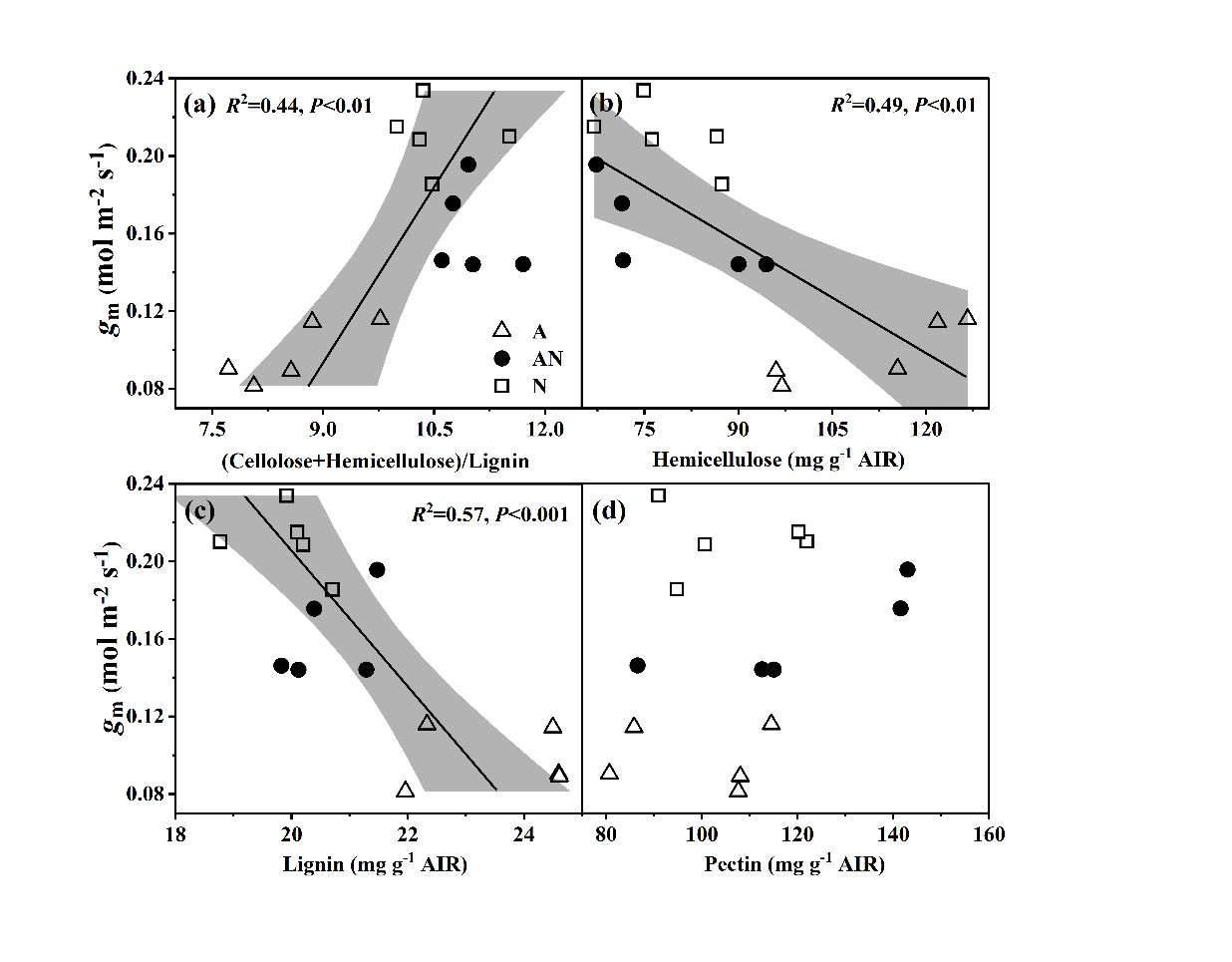


**Fig. S4** Correlations of *g*_m_ and different cell wall components (data obtained from day 28). The amounts of specific cell wall components were expressed based on the amount of AIR. A, AN, and N represent treatments with NH_4_^+^ alone (open triangles), a mixed N supply (closed circles), and NO_3_^–^ alone (open squares), respectively. Data are fitted by linear regression (n=5) and the grey area represents 95% confidence interval.

**
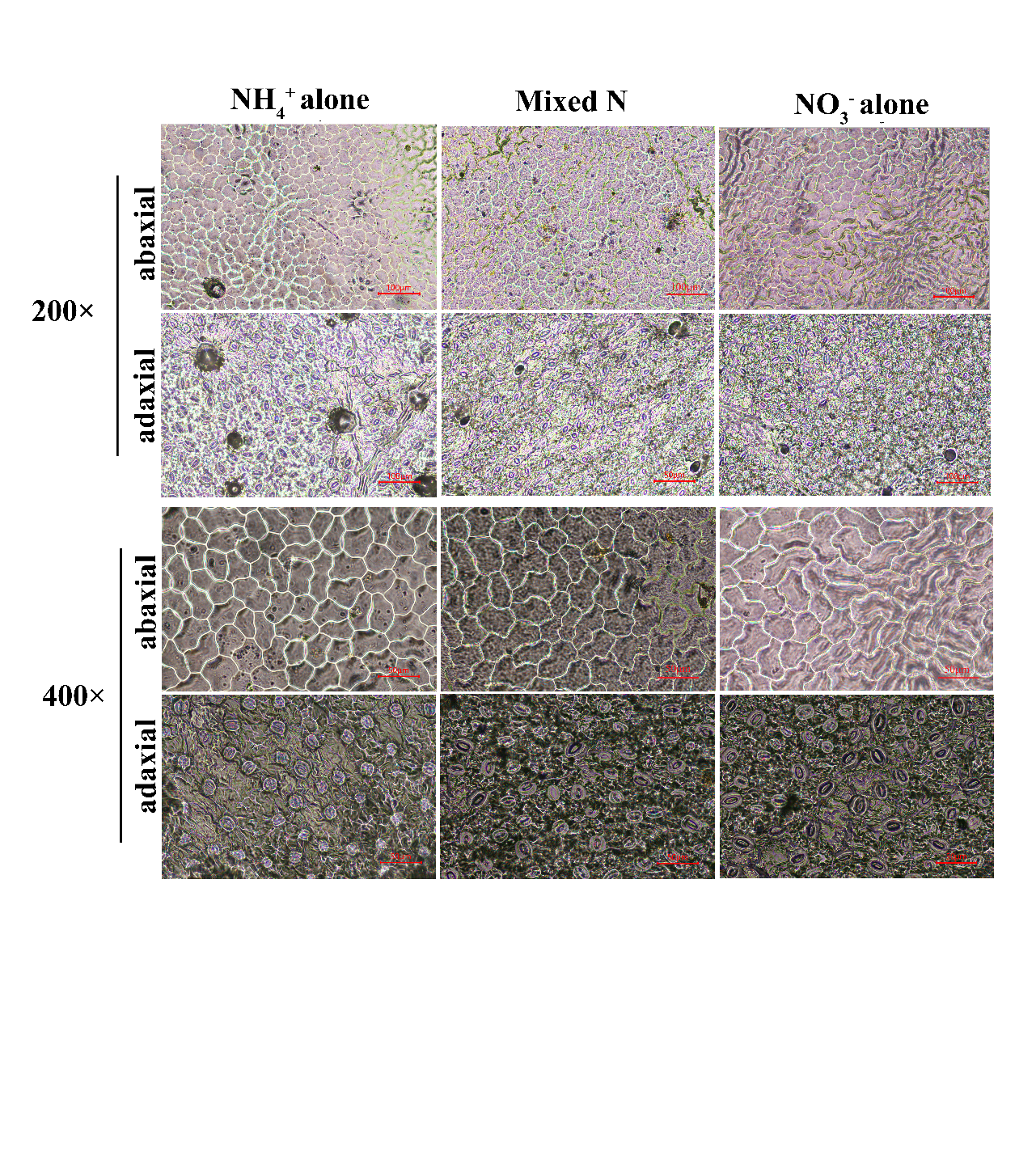
**

**Fig. S5** Effect of different N forms on leaf stomatal microscope characteristics of *L. japonica*.
